# A meta-analysis of four randomized clinical trials to confirm the reliability and responsiveness of the Shortness of Breath with Daily Activities (SOBDA) questionnaire in chronic obstructive pulmonary disease

**DOI:** 10.1186/s12955-015-0369-3

**Published:** 2015-10-31

**Authors:** Maggie Tabberer, Jean Brooks, Teresa Wilcox

**Affiliations:** GSK, Stockley Park West, Uxbridge, Middlesex UB11 1BT UK; Evidera, 7101 Wisconsin Avenue, Suite 1400, Bethesda, MD 20814 USA

**Keywords:** Chronic obstructive pulmonary disease, Dyspnea, Shortness of breath with daily activities questionnaire, SOBDA, COPD, Patient-reported outcome, PRO, Shortness of breath

## Abstract

**Background:**

Chronic obstructive pulmonary disease (COPD) is characterized by non-reversible airflow limitation. A common symptom of COPD is dyspnea or shortness of breath. Dyspnea may vary daily, with a large impact on patients’ lives. Previous clinical trials used patient-reported outcome (PRO) measures that quantified dyspnea at discrete intervals and hence did not reflect this variability. Recently the Shortness of Breath with Daily Activities (SOBDA) questionnaire was developed as a PRO measure of dyspnea utilizing a daily diary. This confirmatory *post hoc* meta-analysis of SOBDA data from a large clinical study program further supports the questionnaire and clarifies the minimum threshold of SOBDA response.

**Methods:**

Data from four clinical trials (DB2113361, NCT01313637; DB2113373, NCT01313650; DB2113360, NCT01316900; DB2113374, NCT01316913) were analyzed. These 24-week trials were randomized, blinded studies investigating the efficacy and safety of several COPD treatments. These *post hoc* analyses focused on the SOBDA questionnaire properties. This electronic-diary consists of 13 items completed daily, in which patients rate their breathlessness level during common daily activities. Resultant SOBDA scores were compared with related, commonly used assessments: modified Medical Research Council Research Dyspnea Scale (mMRC), Baseline Dyspnea Index (BDI), Transition Dyspnea Index (TDI), St George’s Respiratory Questionnaire (SGRQ), COPD Assessment Test (CAT), and trough forced expiratory volume in 1 s (FEV_1_). The consistency, reliability, validity (convergent, known groups), and responsiveness of the SOBDA questionnaire was assessed.

**Results:**

In total, 4967 patients with COPD provided data for these analyses. The SOBDA questionnaire had high internal consistency (Cronbach’s alpha = 0.936), high test-retest reliability (Pearson’s correlation coefficient = 0.86) and convergent validity with related measures (SGRQ total score, Pearson’s correlation coefficient = 0.59; CAT, Spearman rank-order correlation coefficient = 0.50). SOBDA scores were statistically significantly lower in responders (as defined by TDI, SGRQ, CAT, and trough FEV_1_ levels) versus non-responders (*p* < 0.001 for all assessments and all time points). Using an anchor-based method, the threshold of a minimum response was calculated as a SOBDA score change of −0.2 (SOBDA score range = 1–4).

**Conclusions:**

The reliability, validity, and responsiveness of the SOBDA questionnaire as a PRO measure to quantify dyspnea was supported in a large clinical trial population of patients with moderate–very severe COPD.

**Electronic supplementary material:**

The online version of this article (doi:10.1186/s12955-015-0369-3) contains supplementary material, which is available to authorized users.

## Background

Chronic obstructive pulmonary disease (COPD) is characterized by persistent airflow limitation that is not fully reversible [1, 2]. Patients with COPD report dyspnea (also referred to as ‘shortness of breath’ or ‘breathlessness’ by the patient) when undertaking normal physical activities [3], which impacts their health-related quality of life (HRQoL) and often results in medical treatment being sought [2, 4–6]. The relationship between dyspnea and clinical lung function assessment is complex as dyspnea can only be measured from the patient’s perspective [7]. Physiological measures of lung function commonly used in clinical trials, such as the forced expiratory volume in 1 s (FEV_1_; calculated as a percentage of that predicted for a person of the same age gender and height, % predicted FEV_1_), are not always closely related to dyspnea and/or patients’ HRQoL [2, 5]. As dyspnea can vary on a day-to-day basis, the preferred method to assess this symptom is via a daily patient-reported diary [7].

Objectively understanding the efficacy of treatments on dyspnea is critical to ensuring that patients receive optimal care. Even though dyspnea may have a large impact on the daily lives of patients [4], it has been difficult to capture its daily variation within clinical trials [8]. There is currently no available patient-reported outcome (PRO) measure for COPD-related dyspnea that can be used in support of label-specific claims for US Food and Drug Administration (FDA) approved therapies [8]. The Shortness of Breath with Daily Activities (SOBDA) questionnaire was developed to provide such a measure [7–9].

The SOBDA questionnaire enables patients’ perceptions of the impact of dyspnea on their daily activities to be measured and recorded daily in an electronic diary (e-diary). It was developed following guidelines relating to PRO measures from the FDA [9, 10]. Aligned with these guidelines, the SOBDA questionnaire was first developed with data from a cohort of 40 patients with COPD across all stages of disease severity, using focus groups and cognitive debriefing sessions [9]. The usability of the e-diary was assessed in this initial study [9], with the final SODBA questionnaire only available as an e-diary. A larger group of patients (*N* = 334) was involved in a second study designed to test its hypothetical framework and to reduce the number of items within the original 37-item pool [7]. The final 13-item pool was then validated using data from a cohort of patients with moderate–severe COPD (*N* = 547) enrolled in a randomized clinical trial (RCT), to demonstrate the internal consistency, reliability, and validity (convergent and known-groups) of the questionnaire [8]. The next stage, as recommended by the FDA [10], was to further validate the questionnaire within a large group of patients.

This confirmatory *post hoc* meta-analysis measured the performance of the SOBDA questionnaire within a large clinical trial patient population. The specific objectives of these analyses were to a) further assess the reliability and responsiveness of the SOBDA questionnaire using data from four Phase III clinical trials investigating umeclidinium (UMEC)/vilanterol (VI), and b) to refine the SOBDA threshold of minimum response in a clinical trial population.

## Methods

### Study design and patient inclusion criteria

This was a *post hoc* analysis of data (study number 201151) from four clinical trials (DB2113361, NCT01313637; DB2113373, NCT01313650; DB2113360, NCT01316900; DB2113374, NCT01316913) [11–13]. All four trials were 24-week, randomized, blinded, parallel-group studies with similar designs (Additional file 1 and Additional file 2) [11–13]. All patients were required to provide informed consent. The four trials were approved by local institutional review boards/ethics committees [11–13].

Patients were ≥40 years of age, current or former smokers with a smoking history of ≥10 pack-years, and had a physician diagnosis of COPD [1, 2]. Lung function inclusion criteria were a post-albuterol FEV_1_/forced vital capacity ratio <0.70 and a post-albuterol FEV_1_ of ≤70 % of predicted normal. All patients had symptoms of dyspnea at baseline measured using the modified Medical Research Council Dyspnea Scale (mMRC) [14] with a score of at least 2 required. Patients with a current diagnosis of asthma or any clinical significant uncontrolled disease were excluded [11–13]. These inclusion and exclusion criteria were similar to those used in the three SOBDA development studies [7–9], as the SOBDA questionnaire was developed specifically for use in clinical trials of bronchodilators. Patients who met the eligibility criteria at the screening visit completed a 7–10 day and 7–14 day run-in period in DB2113361/DB2113373 [11, 13] and DB2113360/DB2113374 [12] respectively, followed by a 24-week treatment period.

These four trials tested several treatments. Patients were randomized to receive one of the following: placebo, tiotropium 18 mcg, UMEC 62.5 mcg, UMEC 125 mcg, UMEC/VI 62.5/25 mcg, UMEC/VI 125/25 mcg, or VI 25 mcg (the specific treatments used in each trial are detailed in Additional file 1) [11–13]. This *post hoc* analysis focused on the performance of the SOBDA questionnaire and did not investigate the efficacy of these treatments, which have been previously published [11–13]. In these trials, there were a total of ten study clinic visits in DB2113361/DB2113373 [11, 13] and nine visits in DB2113360/DB2113374 [12] (Additional file 2) conducted on an outpatient basis, during which several assessments were made. Patients were asked to complete the SOBDA questionnaire every day throughout the run-in and treatment periods.

### Assessments

To complete the SOBDA questionnaire patients answered 13 items using an e-diary before going to bed each night [7, 9]. Items followed the format of: ‘how short of breath were you when…’ with a list of different daily activities (e.g., ‘…you put on your shoes?’). Response options included a range of four options from ‘not at all’ to ‘so severely that I did not do the activity’, with an additional option of ‘I did not do the activity today’. This last option was included as not all activities were carried out by all patients every day. Therefore, responses were scored either as missing (‘I did not do the activity’) or on a scale ranging from 1 (‘not at all’) to 4 (‘so severely that I did not do the activity’). These responses were used to create a daily mean SOBDA score (mean of the non-missing items, provided at least 7 out of 13 items were non-missing). Aggregated daily scores were converted into weekly SOBDA summary scores only if a daily score was recorded for at least 4 of the 7 days in that week [8]. A low SOBDA score indicated low levels of dyspnea. As this was a multinational study program, the SOBDA questionnaire was developed in US English and translated into 38 languages using translation best practice guidelines [15, 16].

Clinician-rated mMRC was assessed as previously described [12, 14, 17]. Interviewer administered Baseline Dyspnea Index (BDI) and Transition Dyspnea Index (TDI) were assessed using the methods detailed by Mahler et al. [18]. The HRQoL assessment tools, St George’s Respiratory Questionnaire (SGRQ) was completed in all four studies according to Jones et al. [19] and the COPD Assessment Test (CAT) was completed in DB2113360/DB2113374 according to Jones et al. [20]. Spirometry details for measuring trough FEV_1_ were provided in the primary publications relating to the four studies [11–13].

### *Post hoc* analyses

Two study populations were used in these analyses. The run-in population comprised all patients who completed the visit on Day 1, including those who were not randomized, those who were randomized and did not receive any study medication, and those who were randomized and received study medication. The intent-to-treat (ITT) population comprised all randomized patients who received at least one dose of study drug [11–13].

### Consistency, reliability, and validity

The internal consistency of the SOBDA questionnaire was assessed using Cronbach’s alpha (scale from 0 to 1.0, with values ≥0.7 considered as indicative of internal consistency) [21, 22], for all patients in the run-in population who provided a score for each SOBDA item on the first day of the run-in period. Test-retest reliability was assessed in the ITT population for all patients who provided enough data to calculate a SOBDA weekly score at baseline and for the week before Day 28 (SOBDA score recorded on 4 of the 7 days prior to those visits), and had a TDI score of 0 (a surrogate to indicate no change in dyspnea) at the Day 28 visit. Pearson’s correlation and intra-class correlation coefficients (ICC; the threshold goal of >0.60 [23]) were calculated and a t-test used for the comparison of SOBDA baseline score and SOBDA week before Day 28 score.

Convergent validity was assessed for the run-in population by comparing SOBDA baseline score with BDI and SGRQ total score at randomization (Day 1), and baseline trough FEV_1_, using the Pearson’s correlation coefficient. mMRC score at screening (Day −14 to −7) was compared with SOBDA run-in Week 1 score and CAT on Day 1 was compared with SOBDA baseline score using Spearman rank order correlation coefficient.

Known-groups validity was assessed for the run-in population through comparing a) the mean SOBDA baseline score in each BDI category (severity ranged from 0, greatest impairment, to 12, least impairment) as determined at Day 1 (randomization) and b) the SOBDA run-in Week 1 scores in each mMRC category as determined at screening. Both of these were evaluated using analysis of covariance, adjusted for age, gender, geographical region, and percent predicted FEV_1_ at screening. This was repeated excluding percent predicted FEV_1_ due to the high level of data missing for this variable.

### Responsiveness

The responsiveness of the questionnaire was assessed by comparing SOBDA score changes from baseline between responders and non-responders defined by other assessments (TDI, SGRQ, CAT, trough FEV_1_) within the ITT population on Days 28, 84, and 168 (CAT was assessed on Days 84 and 168, according to the study protocols of DB2113360 and DB2113374). The minimum clinically important TDI response was defined as a patient with a TDI focal score of at least 1 unit [11–13]. The minimal clinically important difference (MCID) in SGRQ was defined as a patient with a decrease from baseline (improvement) in SGRQ total score of 4 units or more [11–13] and the MCID in CAT was defined as a patient with a decrease from baseline (improvement) CAT score of 2 units or more [24]. For trough FEV_1_, a patient with a change from baseline of at least 100 mL was considered to be a responder [25].

### Thresholds for SOBDA response

Anchor-based methods and examination of the cumulative proportions of responders and non-responders in the ITT population were used to establish the threshold for SOBDA response [26, 27]. The FDA recommend that the empiric evidence necessary to establish any PRO responder definition is obtained from anchor-based methods, and that the chosen anchors must be easier to interpret than the PRO itself [10]. As such, the three anchors used were: TDI and SGRQ in all four studies at Days 28, 84, and 168, and CAT in Studies DB2113360 and DB2113374 at Days 84 and 168. All three of these measures are widely used to assess patient benefit in COPD clinical trials. In addition to the definitions of minimal responses given above, score changes were assessed in relation to other levels of improvement in the anchor measures (i.e., no change or worse, minor, moderate, major; definitions in Additional file 3). For SGRQ and CAT, a minor improvement was considered to be between one and two-times the MCID (4 units or 2 units, respectively [11–13]), while a moderate improvement was more than twice the MCID. For TDI (where improvements range from 0 to +9 [18]), categories were defined as: minor improvement = 1–3; moderate improvement = 4–6; and major improvement = 7–9. Cumulative distribution plots of change in SOBDA score categorized by each level of improvement were also assessed.

Although they are no longer recommended (by the FDA) as the sole approach to define responders [10], distribution-based methods were used in support of the anchor-based methods, following the methods detailed in Revicki et al. [26] (where 0.2- and 0.3-times the standard deviation [SD] of a PRO was considered to be a measurement of the minimal important difference) and Wyrwich et al. [28] (who used one standard error of the mean [SEM] to assess this) to provide supporting evidence for the responder definition obtained using anchor methods.

## Results

### Patient characteristics

Overall, 4967 patients were included in the run-in population and of these, 4713 patients were included in the ITT population for this *post hoc* meta-analysis. Baseline characteristics and treatment effects for the ITT population were as reported in the primary publications of Studies DB2113360 and DB2113374 by Decramer et al. [12], DB2113361 by Celli et al. [11] and DB2113373 by Donohue et al. [13] (Additional file 1). The mean age of patients in these studies ranged from 62.9 to 64.6 years across all studies and more than 85 % of the patients in each study were Global initiative for Chronic Obstructive Lung Disease (GOLD) Stage II (moderate COPD) or III (severe COPD), less than 15 % were GOLD Stage IV (very severe COPD) and no patients were GOLD Stage I (mild COPD) (Additional file 1).

### Consistency, reliability, and validity

Internal consistency of the SOBDA questionnaire was assessed using Cronbach’s alpha. Within this meta-analysis, Cronbach’s alpha was 0.936 (*n* = 1500: the number of patients with a score for each of the 13 items on the first day of the run-in period, i.e., completed the SOBDA questionnaire daily with no answers of ‘I did not do’).

Test-retest reliability was assessed in the subset of patients who had a SOBDA score at baseline and during the week prior to Day 28, and had a TDI focal score of 0 (a surrogate to indicate no change in dyspnea) at Day 28. SOBDA scores had high test-retest reliability, as indicated by the Pearson’s correlation coefficient of 0.86 and the ICC of 0.85 between SOBDA scores at these two time points. When the test-retest reliability was assessed using a t-test to compare the mean difference between the SOBDA baseline score and that of the week before Day 28 for these patients, the estimated difference was −0.08 (95 % confidence intervals [CI]: −0.10 to −0.06); *n* = 1443). The effect size (difference between the SOBDA baseline score and that of the week before Day 28 divided by the SD of the baseline score) was −0.117.

In the assessment of convergent/divergent validity, SOBDA baseline scores were compared with different assessments for the run-in population. The correlations between SOBDA baseline score and the two HRQoL outcomes (SGRQ total score on Day 1 [Pearson’s correlation coefficient = 0.59; *n* = 4398]; CAT on Day 1 [Spearman rank order correlation coefficient = 0.50; *n* = 1580]) were similar. When the SOBDA baseline score was compared with BDI at Day 1, the correlation was lower than the HRQoL outcomes, (BDI: Pearson’s correlation coefficient = −0.42; *n* = 4495; contrary to SOBDA, higher scores in BDI indicate less dyspnea, hence the negative correlation). Two other correlations, baseline trough FEV_1_ with SOBDA baseline scores (Pearson’s correlation coefficient = −0.17; *n* = 4520) and the clinician-reported mMRC scores at screening with the SOBDA run-in Week 1 scores (Spearman rank order correlation coefficient = 0.32; *n* = 4497) had lower correlations.

To further examine the validity of the SOBDA questionnaire in assessing shortness of breath in patients with COPD, known-groups validity was assessed in the ITT population. The known-groups for this analysis were mMRC scores at screening and BDI scores at Day 1. An association was found between SOBDA run-in Week 1 score and the mMRC scores at screening (F-statistic = 138.52, *p* < 0.001; using an analysis of covariance including all covariates, age, gender and geographical region, % predicted FEV_1_). Similarly an association was found between SOBDA baseline score and BDI score at Day 1 (F-statistic = 45.84, *p* < 0.001; with all covariates). There was a high level of missing data for % predicted FEV_1_ at baseline and when it was excluded as a covariate from this analysis, the known-groups validity F-statistic was 279.49 (*p* < 0.001) for mMRC scores and 83.90 (*p* < 0.001) for the BDI scores (Table 1). Pairwise comparisons of SOBDA scores within each mMRC category showed that the least squares (LS) mean SOBDA score increased as the mMRC increased with all CIs for those comparisons excluding zero. Again, the CIs for the pairwise comparisons of SOBDA scores within each BDI category also excluded zero.

**Table 1:** Known-groups validity: the LS mean SOBDA weekly score by mMRC and by BDI response categories

ANCOVA excluding percent predicted FEV_1_ at screening					
mMRC score^a^ at screening (Day -14 to -7)	mMRC, LS mean run-in Week 1 SOBDA score (SE); n	BDI score at randomization (Day 1)	BDI, LS mean baseline SOBDA score (SE); n	BDI score at randomization (Day 1)	BDI, LS mean baseline SOBDA score (SE); n
0–1	0	0	2.73 (0.108); 32	7	1.84 (0.021); 817
2	1.83 (0.012); 2939	1	2.63 (0.109); 31	8	1.65 (0.029); 434
3	2.25 (0.017); 1395	2	2.57 (0.056); 117	9	1.59 (0.035); 307
4	2.62 (0.050); 163	3	2.48 (0.031); 391	10	1.40 (0.060); 103
		4	2.30 (0.029); 427	11	1.38 (0.115); 28
		5	2.14 (0.027); 499	12	1.49 (0.106); 33
		6	2.00 (0.017); 1276		
Overall F-statistic (p-value)*	279.49 (<0.001)			Overall F-statistic (p-value)*	83.90 (<0.001)

*ANCOVA* analysis of covariance, *BDI* Baseline Dyspnea Index, *FEV*_*1*_ forced expiratory volume in 1 s, *LS* least squares, *mMRC* modified Medical Research Council Dyspnea Scale, *SE* standard error, *SOBDA* Shortness of Breath with Daily Activities

^a^Score 0 = not troubled with breathlessness except during strenuous exercise to 4 = too breathless to leave the house or breathless when dressing or undressing

*ANCOVA adjusted for age, gender, and geographical region (excluding % predicted FEV_1_)

### Responsiveness

**Fig. 1:**
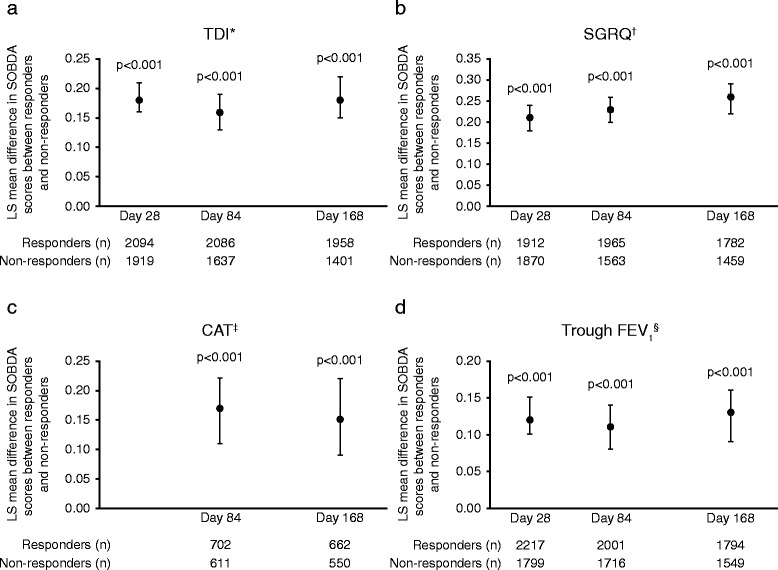
Change in the SOBDA prior week score by TDI (**a**), SGRQ (**b**), CAT (**c**), and trough FEV_1_ (**d**) response. *Response was defined as a TDI focal score of at least 1 unit. ^†^Response was defined as a change from baseline in SGRQ total score of ≤ −4. ^‡^Response was defined as a change from baseline in CAT score of ≤ −2 (studies DB2113360 and DB211374 only). ^§^Response was defined as a change from baseline in trough FEV_1_ of ≥100 mL. Analyses performed using analysis of covariance adjusted for SOBDA baseline score and geographic region. Error bars represent the 95 % CIs. CAT, COPD Assessment Test; CI, confidence interval; COPD, chronic obstructive pulmonary disease; FEV_1_, forced expiratory volume in 1 s; LS, least squares; SGRQ, St George’s Respiratory Questionnaire score; SOBDA, Shortness of Breath with Daily Activities; TDI, Transition Dyspnea Index

To determine if the SOBDA scores were responsive to changes in COPD status as measured by other COPD-related assessments (TDI, SGRQ, CAT, and trough FEV_1_), patients in the ITT population were defined as responders or non-responders according to these other assessments and the SOBDA change scores for these groups were examined. With each of the investigated assessments and time points (TDI, SGRQ, and trough FEV_1_: Day 28, 84, and 168; CAT: Days 84 and 168), LS mean SOBDA change scores were statistically significantly larger in responders compared with non-responders, demonstrating the ability to measure less dyspnea with daily activities (*p* < 0.001 for all assessments and all time points; Fig. 1).

### Thresholds of response using SOBDA scores

The threshold for SOBDA response was assessed using three separate methods in the ITT population. The primary analysis employed anchor-based methods using accepted definitions of MCID for each anchor. Patients who were considered to have improved using TDI, SGRQ, or CAT had decreases in their mean SOBDA score at Day 168 (Table 2) and also at Days 28 and 84 (Additional file 4). These decreases in SOBDA score increased with the level of improvement on the anchor measure. Using these three assessments at Day 168 as anchors, the following thresholds were calculated:

- Change scores associated with minor improvement: −0.16 to −0.22
- Change scores associated with moderate improvement: −0.30 and −0.37
- Change scores associated with major improvement: −0.38 and −0.49

**Table 2:** Thresholds calculated using change from baseline to SOBDA prior week score

	TDI^a^ (Day 168)				SGRQ^b^ (Day 168)			CAT^c^ (Day 168)		
	No change or worse	Minor improvement	Moderate improvement	Major improvement	No change or worse	Minor improvement	Moderate improvement	No change or worse	Minor improvement	Major improvement
n	1375	1108	635	215	1331	437	1345	510	159	503
Mean change from baseline to SOBDA prior week score (SD)	−0.09 (0.515)	−0.22 (0.530)	−0.30 (0.549)	−0.49 (0.574)	−0.02 (0.493)	−0.17 (0.500)	−0.37 (0.547)	−0.10 (0.506)	−0.16 (0.515)	−0.38 (0.608)

*CAT* COPD Assessment Test, *COPD* chronic obstructive pulmonary disease, *SD* standard deviation, *SGRQ* St George’s Respiratory Questionnaire, *SOBDA* Shortness of Breath with Daily Activities, *TDI* Transitional Dyspnea Index

^a^No change or worse is defined as a score of 0 or less, minor improvement is defined as a score of 1–3, moderate improvement is defined as a score of 4–6 and major improvement is defined as a score of 7–9

^b^No change or worse is defined as a change from baseline of > −4 units, minor improvement is defined as a change from baseline of > −8 to ≤ −4 units and moderate improvement is defined as a change from baseline of ≤ −8 units

^c^No change or worse is defined as a change from baseline of > −2 units, minor improvement is defined as a change from baseline of > −4 to ≤ −2 units, major improvement is defined as a change from baseline of ≤ −4 units; for studies DB2113360 and DB2113374

**Fig. 2:**
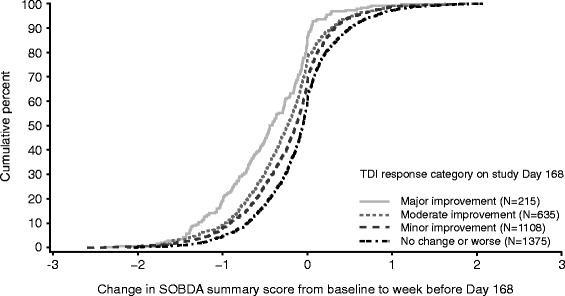
One of the cumulative distribution plots used to calculate SOBDA thresholds. TDI response category definitions: major improvement = score of 7–9, moderate improvement = score of 4–6, minor improvement = score of 1–3, no change or worse = score of 0 or less. SOBDA, Shortness of Breath with Daily Activities; TDI, Transitional Dyspnea Index

Secondly, based on cumulative distribution plots (Fig. 2), the values representing minor improvement for all three anchors fell in a change score range of −0.1 to −0.2 across most time points and supported the calculated values for other levels of improvement.

Finally, with the distribution-based method, response thresholds in SOBDA score were calculated to be −0.135 and −0.203 (0.2 and 0.3 times the SD of the baseline scores, respectively; [26]). According to the SEM method used by Wyrwich et al. [28], the threshold was −0.010. As these scores were based on the SD and SEM, the numbers were calculated as positive numbers. However, within SOBDA scoring, a decrease in the score equates with an improvement in dyspnea, therefore all thresholds of SOBDA scores are negative and these were converted to negative numbers for consistency.

## Discussion

The results of this confirmatory meta-analysis extend the results of previous SOBDA validation studies [7, 8] and demonstrate the consistency, reliability, validity, and responsiveness of the SOBDA questionnaire within a large RCT patient population (*N* = 4967). In addition, the definition of a minimum response threshold for SOBDA score was identified as a decrease from baseline of 0.2 using three separate methods, refining the value within the minimal important difference range of −0.1 to −0.2 previously reported in the smaller SOBDA development study with 547 patients [8].

Within the large patient group used for this meta-analysis, the SOBDA questionnaire was found to demonstrate consistency and test-retest reliability in measuring patient dyspnea with daily activities. The Pearson’s correlation coefficient and the ICC between SOBDA scores in patients with no change in dyspnea, and also Cronbach’s alpha for this meta-analysis, were similar to the two previously published studies using SOBDA [7, 8]. The test-retest reliability was assessed using scores from patients whose clinician endorsed zero for the TDI focal score. Although there was a statistically significant difference in this assessment (*p* < 0.001), which may have been suggestive of a score change, it was likely due to the large patient population studied. The two earlier studies assessed the SOBDA questionnaire in moderate population sizes and found no statistically significant difference between SOBDA scores in the direct measure of patient global assessment of change (PGAC) [7, 8].

The convergent validity correlation between the SOBDA score and SGRQ total score was strong and consistent with a prior SOBDA development study [7]. This was also reflected in the correlation between SOBDA and CAT scores since the SGRQ and CAT measure closely related concepts [20]. While the two previous studies also measured the convergent validity correlation coefficient between the SOBDA score and other dyspnea assessments (PGAC, Clinician Global Impression of Dyspnea-change, Chronic Respiratory Disease Questionnaire- self-administered standardized version) [7, 8], these analyses further determined the convergent validity between SOBDA scores and BDI scores, where there was also a strong correlation. However, the correlation of SOBDA scores with trough FEV_1_ was low, as anticipated due to the weak correlations previously identified between dyspnea and trough FEV_1_ [29, 30]. Also, there was a weak correlation between SOBDA score and mMRC, previously found by Watkins et al. who suggested that this was due to the narrow range of responses given by clinicians when measuring mMRC [8]*.* The SOBDA questionnaire, therefore, appears to correlate better with other patient completed measures that provide stratification in levels of response, rather than measures administered by an interviewer or clinician. Within the current meta-analysis, SOBDA scores were shown to have known-groups validity based on the clinician-rated mMRC and interviewer-administered BDI. The two prior studies demonstrated that this was also the case for patient-completed mMRC and the clinician-completed global impression of symptoms [7, 8].

This *post hoc* analysis was also conducted to confirm a response threshold using an anchor-based and supportive distribution-based approach. Previous studies suggested a threshold for response in the range of −0.1 to −0.2 (where SOBDA weekly scores ranged from 1 to 4). Both the anchor-based and distribution-based methods in this study support a −0.2 threshold of minimum response. Thresholds for moderate and major improvement were not formally assessed, but the data suggest that a threshold for moderate improvement would be a SOBDA score change of −0.30 to −0.37 and for major improvement would be a SOBDA score change of −0.38 to −0.49. These results are in agreement with the review by Revicki et al., wherein the authors stated that the anchor-based methods are recommended, but the distribution methods may provide guidance [26]. Revicki et al. considered the differences between using 0.5, 0.3 and 0.2 times the SD as the minimal difference and concluded that the 0.5 times the SD was likely too large to be the minimal non-ignorable difference.

When considering missing data we found that completion of the SOBDA daily e-diary, measured by data transmission, exceeded 90 % indicating that patients were able to use the e-diary over an extended study period. There were no important gender or country differences in the incidence of ‘I did not do this activity today’ responses for individual SOBDA questionnaire items, indicating that the final item pool is appropriate for both genders and across multiple countries (GSK, data on file).

This meta-analysis of SOBDA data had its limitations. Due to the inclusion criteria used, the majority of patients in this meta-analysis were at GOLD Stage II or III at baseline (Additional file 1), with approximately 10 % (*n* = 494) at GOLD Stage IV. Future investigations into the SOBDA questionnaire could involve a more detailed analysis of this GOLD Stage IV subgroup and also studies to include those at the very early stages of this disease. The results presented here are applicable to a RCT population with moderate–very severe COPD. It remains unclear how the SOBDA questionnaire would perform in the overall COPD population or in an exacerbating COPD population, particularly as patients were withdrawn from these studies if they had an exacerbation.

The SOBDA questionnaire was developed as a daily e-diary for use in clinical research, rather than for use in clinical practice where the patient burden of completing a daily diary is likely to be too onerous. Through its use within clinical trials, the SOBDA questionnaire may be able to contribute to the assessment of new therapies that improve dyspnea in patients with COPD.

## Conclusions

The findings of this meta-analysis confirm and extend the results of previous SOBDA validation studies, and demonstrate the consistency, reliability, and validity of the SOBDA questionnaire under RCT conditions. A minimum threshold of response in SOBDA score was confirmed as a change of −0.20. With support for the SOBDA questionnaire proven to be consistent, valid, and reliable in patients with moderate–very severe COPD, this response threshold could be incorporated into the assessments used in future RCTs of COPD therapies.

## Additional files

Additional file 1:
**Design of the four studies and patient baseline characteristics.** (DOC 64 kb) Additional file 2:
**Schematic of the study design used in each of the four included studies.** *Studies DB2113361 [11] and DB2113373 [13]. ^†^Studies DB2113360 (Study 1) and DB2113374 (Study 2) [12]. Baseline SOBDA score refers to data collected during the 7 days prior to V2. SOBDA, Shortness of Breath with Daily Activities; V, visit. (PDF 990 kb) Additional file 3:
**Definitions of the levels of improvement used for analysis.** (DOC 29 kb) Additional file 4:
**Thresholds calculated using change from baseline to SOBDA prior week score at Days 28 and 84.** (DOC 31 kb)

## Abbreviations

BDI: Baseline Dyspnea Index
CAT: COPD Assessment Test
CI: Confidence interval
COPD: Chronic obstructive pulmonary disease
e-diary: Electronic diary
FDA: Food and Drug Administration
FEV_1_: Forced expiratory volume in 1 s
GOLD: Global initiative for chronic Obstructive Lung Disease
HRQoL: Health-related quality of life
ICC: Intra-class correlation
ITT: Intent-to-treat
LS: Least squares
MCID: Minimal clinically important difference
mMRC: Modified Medical Research Council Dyspnea Scale
PGAC: Patient global assessment of change
PRO: Patient-reported outcome
RCT: Randomized clinical trial
SD: Standard deviation
SEM: Standard error of the mean
SGRQ: St George’s respiratory Questionnaire
SOBDA: Shortness of Breath with Daily Activities
TDI: Transition Dyspnea Index
UMEC: Umeclidinium
V: Visit
VI: Vilanterol
