# Supplementary material for: A meta-analysis of four randomized clinical trials to confirm the reliability and responsiveness of the Shortness of Breath with Daily Activities (SOBDA) questionnaire in chronic obstructive pulmonary disease
Source: Health Qual Life Outcomes. 2015 Oct 31;13:177. doi: 10.1186/s12955-015-0369-3 (PMC4628367; doi:10.1186/s12955-015-0369-3)
Supplement: Additional file 1: — Design of the four studies and patient baseline characteristics. (DOC 64 kb) [file 12955_2015_369_MOESM1_ESM.doc]

**Additional file 1. Design of the four studies and patient baseline characteristics**

| **Characteristic** | **Decramer *et al.* DB2113360 (Study 1)** | **Decramer *et al.***  **DB2113374 (Study 2)** | **Celli *et al.***  **DB2113361** | **Donohue *et al.***  **DB2113373** |
| --- | --- | --- | --- | --- |
| Patients randomized | 846 (1:1:1:1, across four treatment arms) | 872 (1:1:1:1, across four treatment arms) | 1493 (3:2, active treatments: placebo) | 1536 (3:2, active treatments: placebo ) |
| Treatment arms | 1) UMEC/VI 125/25 mcg  2) UMEC/VI 62.5/25 mcg  3) TIO 18 mcg  4) VI 25 mcg (Study 1) or UMEC 125 mcg (Study 2) | | 1) UMEC/VI 125/25 mcg  2) UMEC 125 mcg  3) VI 25 mcg  4) Placebo | 1) UMEC/VI 62.5/25 mcg  2) UMEC 62.5 mcg  3) VI 25 mcg  4) Placebo |
| Patients within the ITT population | Total: 843; however, 823 used in the final analysis* | Total: 869 | Total: 1489 | Total: 1532 |
| Availability of CAT data | Available | Available | Not available | Not available |
| ***Demographics of the ITT population*** | | | | |
| Mean age in years (SD) | 62.9 (9.0) | 64.6 (8.4) | 62.9 (8.5) | 63.1 (8.9) |
| Female, n (%) | 261 (31) | 280 (32) | 515 (35) | 449 (29) |
| Race |  |  |  |  |
| White, n (%) | 723 (86) | 657 (76) | 1314 (88) | 1303 (85) |
| African American/African heritage, n (%) | 25 (3) | 31 (3) | 24 (2) | 47 (3) |
| Other, n (%) | 95 (11) | 181 (21) | 151 (10) | 182 (12) |
|  |  |  |  |  |
| GOLD Stage [% predicted FEV_1_] at screening, n | 835 | 865 | 1483 | 1529 |
| Stage I [≥80%], n (%) | 0 | 0 | 0 | 0 |
| Stage II [≥50% to <80%], n (%) | 393 (47) | 384 (44) | 699 (47) | 708 (46) |
| Stage III [≥30% to <50%], n (%) | 350 (42) | 374 (43) | 660 (45) | 650 (43) |
| Stage IV [<30%], n (%) | 92 (11) | 107 (12) | 124 (8) | 171 (11) |

*20 patients were excluded from the efficacy analysis due to the lack of adherence of one investigator to good clinical practice.

CAT, COPD Assessment Test; COPD, chronic pulmonary obstructive disease; FEV_1_, forced expiratory volume in 1 second; GOLD, Global initiative for chronic Obstructive Lung Disease; ITT, intent-to-treat; SD, standard deviation; TIO, tiotropium; UMEC, umeclidinium; VI, vilanterol.
