# Supplementary figures and images for: A meta-analysis of four randomized clinical trials to confirm the reliability and responsiveness of the Shortness of Breath with Daily Activities (SOBDA) questionnaire in chronic obstructive pulmonary disease
Source: Health Qual Life Outcomes. 2015 Oct 31;13:177. doi: 10.1186/s12955-015-0369-3 (PMC4628367; doi:10.1186/s12955-015-0369-3)

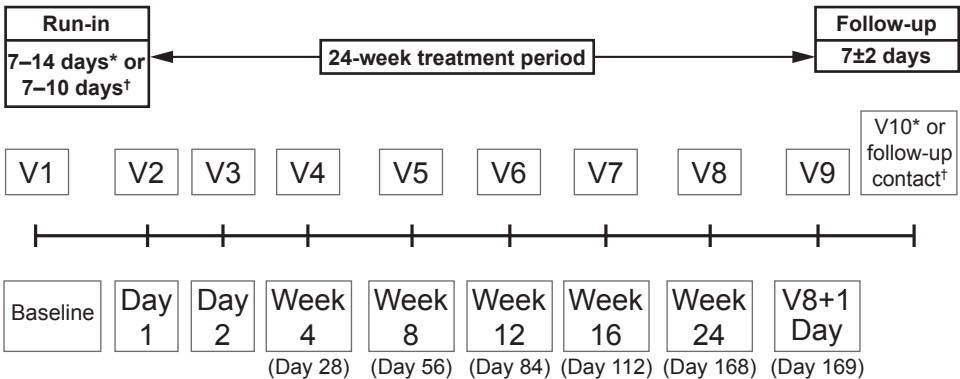

Supplement: Additional file 2: — Schematic of the study design used in each of the four included studies. *Studies DB2113361 [11] and DB2113373 [13]. †Studies DB2113360 (Study 1) and DB2113374 (Study 2) [12]. Baseline SOBDA score refers to data collected during the 7 days prior to V2. SOBDA, Shortness of Breath with Daily Activities; V, visit. (PDF 990 kb) [file 12955_2015_369_MOESM2_ESM.pdf]
