# Supplementary material for: A meta-analysis of four randomized clinical trials to confirm the reliability and responsiveness of the Shortness of Breath with Daily Activities (SOBDA) questionnaire in chronic obstructive pulmonary disease
Source: Health Qual Life Outcomes. 2015 Oct 31;13:177. doi: 10.1186/s12955-015-0369-3 (PMC4628367; doi:10.1186/s12955-015-0369-3)
Supplement: Additional file 3: — Definitions of the levels of improvement used for analysis. (DOC 29 kb) [file 12955_2015_369_MOESM3_ESM.doc]

**Additional file 3.** Definitions of the levels of improvement used for analysis

| **Score (units)** | **No change or worse** | **Minor improvement** | **Moderate improvement** | **Major improvement** |
| --- | --- | --- | --- | --- |
| TDI | 0 or less | 1–3 | 4–6 | 7–9 |
| SGRQ | >-4 | >-8 to ≤-4 | ≤-8 | Not used |
| CAT | >-2 | >-4 to ≤-2 | ≤-4 | Not used |

CAT, COPD Assessment Test; COPD, chronic pulmonary obstructive disease; SGRQ, St George’s Respiratory Questionnaire; TDI, Transitional Dyspnea Index.
