# Supplementary material for: A meta-analysis of four randomized clinical trials to confirm the reliability and responsiveness of the Shortness of Breath with Daily Activities (SOBDA) questionnaire in chronic obstructive pulmonary disease
Source: Health Qual Life Outcomes. 2015 Oct 31;13:177. doi: 10.1186/s12955-015-0369-3 (PMC4628367; doi:10.1186/s12955-015-0369-3)
Supplement: Additional file 4: — Thresholds calculated using change from baseline to SOBDA prior week score at Days 28 and 84. (DOC 31 kb) [file 12955_2015_369_MOESM4_ESM.doc]

**Additional file 4.** Thresholds calculated using change from baseline to SOBDA prior week score at Days 28 and 84

|  | **TDI^*^ (Day 28)** | | | | **SGRQ^†^ (Day 28)** | | | **CAT^‡^ (Day 28)** | | |
| --- | --- | --- | --- | --- | --- | --- | --- | --- | --- | --- |
|  | **No change or worse** | **Minor improvement** | **Moderate improvement** | **Major improvement** | **No change or worse** | **Minor improvement** | **Moderate improvement** | **No change or worse** | **Minor improvement** | **Major improvement** |
| n | 1919 | 1400 | 562 | 132 | 1870 | 570 | 1342 | (CAT data at Day 28 were not collected) | | |
| Mean change from baseline to SOBDA prior week score (SD) | -0.07  (0.416) | -0.20  (0.427) | -0.32  (0.464) | -0.51  (0.520) | -0.05  (0.389) | -0.16  (0.383) | -0.32  (0.477) |
|  | **TDI^*^ (Day 84)** | | | | **SGRQ^†^ (Day 84)** | | | **CAT^‡^ (Day 84)** | | |
| n | 1618 | 1263 | 655 | 168 | 1517 | 462 | 1503 | 569 | 190 | 512 |
| Mean change from baseline to SOBDA prior week score (SD) | -0.10  (0.486) | -0.19  (0.469) | -0.32  (0.530) | -0.44  (0.566) | -0.04  (0.447) | -0.17  (0.434) | -0.33  (0.532) | -0.08  (0.465) | -0.21  (0.514) | -0.32 (0.550) |

^*^No change or worse is defined as a score of 0 or less, minor improvement is defined as a score of 1-3, moderate improvement is defined as a score of 4–6 and major improvement is defined as a score of 7–9.

**^†^**No change or worse is defined as a change from baseline of >-4 units, minor improvement is defined as a change from baseline of >-8 to ≤-4 units and moderate improvement is defined as a change from baseline of ≤-8 units.

**^‡^**No change or worse is defined as a change from baseline of >-2 units, minor improvement is defined as a change from baseline of >-4 to ≤-2 units, major improvement is defined as a change from baseline of ≤-4 units; for studies DB2113360 and DB2113374.

CAT, COPD assessment test; COPD, chronic pulmonary obstructive disease; SD, standard deviation; SGRQ, St George’s Respiratory Questionnaire; SOBDA, Shortness of Breath with Daily Activities; TDI, Transitional Dyspnea Index.
